# Diagnostic approach in patients with angina and no obstructive coronary artery disease: emphasising the role of the coronary function test

**DOI:** 10.1007/s12471-020-01532-9

**Published:** 2021-01-07

**Authors:** R. E. Konst, P. Damman, D. Pellegrini, N. van Royen, A. H. E. M. Maas, S. E. Elias-Smale

**Affiliations:** 1 Department of Cardiology, Radboud University Medical Center, Nijmegen, The Netherlands; 2 University of Milano-Bicocca, Milan, Italy

**Keywords:** Microvascular angina, Coronary vasospasm, Coronary function testing, Coronary angiography, Coronary vascular dysfunction

## Abstract

**Background:**

Many patients with angina do not have obstructive coronary artery disease (CAD), also referred to as “Ischaemia with No Obstructive Coronary Arteries“ (INOCA). Coronary vascular dysfunction is the underlying cause of this ischaemic heart disease in as much as 59–89% of these patients, including the endotypes of coronary microvascular dysfunction and epicardial coronary vasospasm. Currently, a coronary function test (CFT) is the only comprehensive diagnostic modality to evaluate all endotypes of coronary vascular dysfunction in patients with INOCA.

**Objective:**

In this paper we discuss the relevance of performing a CFT, provide considerations for patient selection, and present an overview of the procedure and its safety.

**Methods:**

We reviewed the latest published data, guidelines and consensus documents, combined with a discussion of novel original data, to present this point of view.

**Results:**

The use of a CFT could lead to a more accurate and timely diagnosis of vascular dysfunction, identifies patients at risk for cardiovascular events, and enables stratified treatment which improves symptoms and quality of life. Current guidelines recommend considering a CFT in patients with INOCA and persistent symptoms. The safety of the procedure is comparable to that of a regular coronary angiography with physiological measurements. Non-invasive alternatives have limited diagnostic accuracy for the identification of coronary vascular dysfunction in patients with INOCA, and a regular coronary angiography and/or coronary computed tomography scan cannot establish the diagnosis.

**Conclusions:**

A complete CFT, including acetylcholine and adenosine tests, should be considered in patients with INOCA.

**Supplementary Information:**

The online version of this article (10.1007/s12471-020-01532-9) contains supplementary material, which is available to authorized users.

## Introduction

Obstructive coronary artery disease (CAD) is the best-known cause of ischaemic heart disease (IHD). However, up to two thirds of women and one third of men undergoing coronary angiography for suspected stable IHD have no or non-obstructive CAD [1, 2]. This is also referred to as Ischaemia with No Obstructive Coronary Arteries (INOCA). Patients with INOCA have either signs or symptoms of IHD, such as regional reversible ischaemia on functional testing, or chest pain or dyspnoea. Coronary vascular dysfunction is the underlying cause of INOCA in up to almost 90% of patients [3–6], encompassing the endotypes of coronary microvascular dysfunction (CMD) and epicardial coronary vasospasm [7–9]. Coronary vascular dysfunction can also occur in patients with a history of obstructive CAD [9].

Coronary vascular dysfunction is often not recognised, despite the diagnostic criteria for both the endotype of epicardial spasm and of CMD provided by the Coronary Vasomotor Disorder International Study group (COVADIS), as summarised in Tab. 1 [4, 5]. Data clearly indicates that not properly diagnosing coronary vascular dysfunction deprives patients of a tailored treatment and is associated with persistent symptoms, impaired quality of life, repeated hospitalisations, and unnecessary diagnostic procedures in search of obstructive CAD [2, 9–11]. The key to a definite diagnosis is to demonstrate a dysfunction of the coronary arteries, which cannot be assessed completely using solely non-invasive methods [9, 12]. Therefore, the current reference standard for the diagnosis of coronary vascular dysfunction is an invasive coronary function test (CFT), performed during a coronary angiography [9, 12]. In contrast to Asia, CFT is not yet widely implemented in Europe and the United States [7]. At the Radboud University Medical Center in Nijmegen, the Netherlands, we have been performing CFT since 2019.

**Table 1:** Diagnostic criteria for vasospastic angina and microvascular angina

	Criteria for vasospastic angina	Criteria for microvascular angina
Symptoms	1. Nitrate-responsive angina—during spontaneous episode, with at least one of the following: a. Angina at rest—especially between night and early morning b. Marked diurnal variation in exercise tolerance—reduced in morning c. Hyperventilation can precipitate an episode d. Calcium channel blockers (but not beta blockers) suppress episodes	1. Symptoms of myocardial ischaemia a. Angina on effort or at rest b. Angina equivalents (i.e. shortness of breath)
Absence of obstructive CAD	*Not mentioned*	2. Absence of obstructive CAD (> 50% diameter reduction or FFR < 0.80) by a. Coronary CTA b. Invasive coronary angiography
Myocardial ischaemia	2. Transient ischaemic ECG changes during spontaneous episode, including any of the following in at least two contiguous leads: a. ST-segment elevation ≥ 0.1 mV b. ST-segment depression ≥ 0.1 mV c. New negative U waves	3. Objective evidence of myocardial ischaemia a. Ischaemic ECG changes during an episode of chest pain b. Stress-induced chest pain and/or ischaemic ECG changes in the presence or absence of transient/reversible abnormal myocardial perfusion and/or wall motion abnormality
Impaired vascular function	3. Coronary artery spasm—defined as transient total or subtotal coronary artery occlusion (> 90% constriction) with angina and ischaemic ECG changes either spontaneously or in response to a provocative stimulus (typically acetylcholine, ergot, or hyperventilation)	4. Evidence of impaired coronary microvascular function a. Impaired coronary flow reserve (cut-off values between ≤ 2.0 and ≤ 2.5 depending on used methodology) b. Coronary microvascular spasm, defined as reproduction of symptoms, ischaemic ECG shifts but no epicardial spasm during acetylcholine testing. c. Abnormal coronary microvascular resistance indices (e.g. IMR > 25) d. Coronary slow flow phenomenon, defined as TIMI frame count < 25
Definite diagnosis	Nitrate-responsive angina is evident during spontaneous episodes and either the transient ischaemic ECG changes during the spontaneous episodes or coronary artery spasm criteria are fulfilled	All four criteria are present for a diagnosis of microvascular angina
Suspected diagnosis	Nitrate-responsive angina is evident during spontaneous episodes but transient ischaemic ECG changes are equivocal or unavailable and coronary artery spasm criteria are equivocal	Criteria 1 and 2 are present with either criteria 3 or criteria 4

*ECG* electrocardiographic, *CAD* coronary artery disease, *CTA* computed tomography angiography, *FFR* fractional flow reserve, *IMR* index of microvascular resistance, *TIMI *thrombolysis in myocardial infarction

In this point-of-view paper we will discuss the importance of establishing the diagnosis of coronary vascular dysfunction in patients with INOCA, for which we will focus on the invasive CFT. We will provide considerations for patient selection and share our experiences with the procedure and its safety. We will shortly discuss alternative non-invasive diagnostic modalities that can be used to diagnose coronary vascular dysfunction in patients with INOCA [9]. This paper will discuss only patients with stable IHD, and will not elaborate on patients who suffered a myocardial infarction with non-obstructive coronary arteries (MINOCA) since a recent position paper from the Dutch ACS working group already highlights this group of patients in great detail [13].

## Relevance of diagnosing coronary vascular dysfunction

It should be stressed that INOCA is a working diagnosis, and should prompt physicians to consider additional testing to distinguish the underlying diagnosis of coronary vascular dysfunction from non-cardiac conditions that can present as INOCA [9, 14, 15]. Diagnosing coronary vascular dysfunction is relevant to the identification of patients who are at high risk, [10] to tailored treatment and improvement of symptoms and quality of life and [17] to the potential prevention of unnecessary diagnostic procedures [9].

First, the presence of coronary vascular dysfunction is a consistent and independent predictor of adverse cardiovascular events [10]. The endotype of microvascular dysfunction (low coronary flow and/or high microvascular resistance) is especially associated with cardiovascular death, myocardial infarction, stroke and heart failure [10]. The endotype of epicardial spasm predicts future myocardial infarction and repeat angiography, while the endotype of microvascular spasm are at higher risk for recurrent angina [16]. The combination of epicardial vasospasm and microvascular dysfunction (abnormal index of microvascular resistance—IMR) has recently been shown to be an especially high-risk endotype of coronary vascular dysfunction, with high rates of cardiac death, myocardial infarction and hospitalisation for unstable angina [5, 17]. If a CFT is performed, it should therefore be encouraged to assess all endotypes with both acetylcholine and adenosine testing.

Second, the diagnosis of the endotype of vascular dysfunction enables stratified treatment, and leads to a significant improvement in angina and quality of life. The CorMiCa trial has shown this in 151 patients with INOCA: patients who received treatment that was stratified according to the CFT-diagnosed endotype of coronary vascular dysfunction experienced less angina and an improved quality of life compared with patients who received standard care [17]. The most recent follow-up data show that this improvement is sustained for at least a year [17].

Third, performing a CFT could help prevent unnecessary repeat diagnostic testing in search of obstructive CAD in patients with INOCA. The Radboud University Medical Center features a specialised outpatient clinic for patients with INOCA. Prior to performing CFTs in our centre, we assessed the amount of excess diagnostic testing that patients with INOCA underwent before they were referred to our centre by a systematic study of the patient records of referred patients. This number would help us to identify the need for a single diagnostic test that can establish the diagnosis of coronary vascular dysfunction in patients with INOCA. Between 2015 and 2017 we identified 206 consecutive patients with INOCA and suspected vascular dysfunction (mean age 56 ± 8.6 years and 96% female), referred to our centre. The complete overview of the patient characteristics is included in supplementary Table 1. Patients had a median angina duration of 1.8 years (ranging from 1 month to 18.7 years), and underwent a median of 4 diagnostic tests (ranging from 0 to 13 tests) before referral. As seen in supplementary Table 1, 45% of these patients underwent more than one anatomical coronary angiography and/or coronary computed tomography scan, whereas one third of the patients underwent more than one functional non-invasive ischaemia detection test. These data represent a selected group of patients with chronic angina referred for a second opinion. However, the amount of unnecessary repeat diagnostic testing is considerable. Future studies should shed light on the cost-effectiveness of performing a CFT as soon as obstructive CAD is ruled out with either anatomical or functional diagnostic imaging.

## Patient selection

When a CFT is performed in patients with INOCA, coronary vascular dysfunction is present in up to 89% of patients [3–5]. The majority of these patients have epicardial or microvascular spasm, whereas impairment of other measures of microvascular function, such as IMR and coronary flow reserve (CFR), is less frequently observed [3, 18, 19]. Independent predictors of coronary vascular dysfunction are female sex, a history of obstructive CAD, and a clinical presentation with exercise-induced angina or both exercise-induced angina and angina at rest [3].

Since CFT is not widely available, it is important to select the patients who likely benefit most from this test. Lifestyle management and an empirical treatment with anti-anginal agents should be initiated first in patients with suspected vascular dysfunction [9, 12]. Also, even though the risk of an invasive CFT is not higher than the risk of a regular coronary angiography, including physiological measurements with the use of an intracoronary wire, this risk should always be weighed against the potential benefits.

It should be stressed that the selection of patients for and the timing of a CFT is still under active investigation. Future studies should point out whether we should consider a CFT more liberally in all patients with INOCA as soon as obstructive CAD is ruled out, or whether it should be used more selectively, e.g. in patients with persistent symptoms. Which of these strategies is most beneficial for symptom management and quality of life, prognosis but also cost-effectiveness, is currently unclear. In our opinion, with current evidence and in line with current Dutch recommendations [15], performing a CFT should be considered in the following situations:

- If establishing a definite diagnosis of vascular dysfunction is important for the treating physician, for example to aid tailored treatment when different anti-anginal agents are unsuccessful in improving the anginal symptoms in the patient with INOCA [17]. A CFT should also be considered for risk assessment or when it is important to rule-out vascular dysfunction as a cause of the symptoms.
- If establishing a definite diagnosis of vascular dysfunction is important for the patient with INOCA, for example to gain clarity regarding the diagnosis of vascular dysfunction, as aid in acceptation of the disease, or in the setting of a disability assessment procedure.

## How to perform coronary reactivity testing

A CFT consists of a standard diagnostic coronary angiography, including intracoronary physiological assessment to rule out obstructive CAD if intermediate stenoses are seen, followed by a comprehensive physiological assessment to evaluate macro- and microvascular function. Patients should withhold all vasoactive medication and methylxanthine-containing agents at least 24–48 h prior to scheduled testing (depending on half-life), and should refrain from smoking and short-acting nitroglycerin with a minimum of 4 h before the procedure [20].

### Endothelium-dependent macro- and microvascular vasoreactivity: spasm provocation

Intracoronary acetylcholine is used to assess endothelium-dependent coronary vascular function. Under physiological circumstances, administration of acetylcholine leads to a net vasodilation effect [21]. If endothelial dysfunction is present, vasodilation is impaired causing a net vasoconstriction [21]. To test this, incremental doses of 2, 20, 100, and 200 mcg are manually infused in 1–3 min into the left coronary artery (LCA) via a coronary catheter, under continuous monitoring of heart rate, blood pressure and a 12-lead electrocardiogram. After each infusion dose a repeat angiogram is made to determine the change in coronary diameter. The occurrence of anginal symptoms and ischaemic electrocardiographic changes (defined as transient ST-segment depression or elevation ≥ 0.1 mV in at least 2 contiguous leads) are noted. If a luminal diameter reduction of > 90% occurs (either diffuse or focal), epicardial vasospasm is diagnosed. If symptoms and ischaemic electrocardiographic changes occur, but the luminal reduction is ≤ 90%, the diagnosis microvascular spasm is made (Tab. 2). After spasm occurs, or if the maximum 200 mcg dose is completed, intracoronary nitroglycerin is administered. In patients with no signs of spasm in the LCA and a high likelihood for spasm, one may consider testing the vasoreactivity of the right coronary artery (RCA), additionally using 80 mcg of acetylcholine.

**Table 2:** Definition of spasm during acetylcholine provocation testing

	Recognisable symptoms	Ischaemic ECG changes	≥ 90% diameter reduction^a^
*Epicardial spasm*	Yes	Yes	Yes
*Microvascular spasm*	Yes	Yes	No
*No spasm*	No	No	No
*Inconclusive*	All other combinations		

*ECG* electrocardiographic

^a^ Epicardial coronary diameter reduction ≥ 90% compared with the relaxed state following intracoronary nitroglycerin infusion

### Non-endothelium-dependent vasoreactivity: nitroglycerin and adenosine

Nitroglycerin predominantly dilates the epicardial coronary arteries and represents non-endothelium-dependent epicardial reactivity [22]. Adenosine predominantly dilates the coronary microcirculation [23]. Intact endothelium does not seem to be necessary for an adenosine response, making it also mainly a non-endothelium-dependent reaction [23]. Adenosine is used to measure the coronary flow reserve (CFR) and the microvascular resistance. We generally use the thermodilution method to acquire these measurements [24]. First, a guidewire with distal pressure and temperature sensors (PressureWire X, Abbott Vascular, Santa Clara, CA, USA) is positioned in a distal coronary artery (preferably the left anterior descending artery). Then, resting mean transit time (Tmn) is measured by injections of 3 ml saline at room temperature. The aortic pressure at the guiding catheter (Pa) and the distal coronary pressure at the tip of the guidewire (Pd) are recorded simultaneously. Subsequently, measurements are repeated during maximal hyperaemia—and thereby minimal microvascular resistance—using 140 mcg/kg/min intravenous adenosine. The index of microvascular resistance (IMR) is calculated as the Pd at maximal hyperaemia divided by the inverse of the hyperaemic Tmn [25]. CFR is calculated as the average resting Tmn divided by the average hyperaemic Tmn, averaging at least three consecutive overlapping measurements [26]. CMD is defined as a CFR below 2.0 (or 2.5, depending on measurement methodology) and/or an IMR > 25 (see Tab. 1; [8]). Alternatively, CFR and microvascular resistance (hyperaemic microvascular resistance) can be measured with use of a Doppler flow wire. The Doppler flow method is less feasible because it can be challenging to obtain a stable Doppler flow signal.

## Safety of the coronary function test

CFT has a comparable safety profile with coronary angiography and related procedures. Serious complications such as coronary artery dissection, myocardial infarction and ventricular arrhythmias occur in less than 1% as shown in large cohorts of patients in both Asia and other continents [20, 27, 28]. The most common minor complications are paroxysmal atrial fibrillation (prevalence of 1.6%), and transient atrioventricular block (prevalence 2.2%) during the injection of acetylcholine (especially in the RCA). The latter often resolves after reducing the speed of the manual infusion [27]. Essential to this low complication rate is an experienced and dedicated interventional team which is familiar with the protocols and techniques [20].

## Non-invasive alternatives to diagnose coronary vascular dysfunction

It is beyond the scope of this point-of-view paper to provide an in-depth overview of non-invasive modalities for the diagnosis of coronary vascular dysfunction, as it has already been provided by others [9, 12, 29]. We do, however, want to touch upon the available alternatives that currently include Doppler echocardiography, cardiac magnetic resonance imaging, and—the most validated and accurate—positron emission tomography [29]. Besides their limited availability, the major shortcoming of these non-invasive modalities is that they generally only assess the CFR or variants thereof. They thereby fail to detect coronary spasms, while coronary spasms are the most frequent endotype of coronary vascular dysfunction. Non-invasive methods that assess coronary spams are available, for example the implementation of mental stress, hyperventilation or the cold-pressor technique to provoke spasm. However, besides the lack of experience in most centres, there is little data about the validity and safety of these methods [30].

## Conclusions and future perspectives

The key message is that an invasive CFT is a feasible, useful and safe method to identify coronary vascular dysfunction in patients with INOCA. Within the last years, several Dutch cardiology centres have started to perform CFTs, making this modality more widely available. The exact role of CFT in daily practice, including the best selection strategy, should be determined in collaboration with all expertise centres and a sharing of knowledge and expertise.

Future studies should enhance our understanding of the underlying pathophysiology of coronary vascular dysfunction, provide us with more prospective data regarding the specific outcomes of the various endotypes, and should elaborate on the best patient selection strategy, including cost-effectiveness and timing of the CFT in patients with INOCA. Collaborative prospective registries should be initiated with the hospitals that perform CFT to enable pooled analyses and create a platform for expertise and studies that focus on treatment.

## Supplementary Information

Supplementary Table 1: Characteristics of consecutive patients with INOCA referred to the specialised outpatient clinic
