# Supplementary material for: Diagnostic approach in patients with angina and no obstructive coronary artery disease: emphasising the role of the coronary function test
Source: Neth Heart J. 2021 Jan 7;29(3):121–8. doi: 10.1007/s12471-020-01532-9 (PMC7904984; doi:10.1007/s12471-020-01532-9)
Supplement: Supplementary file 1 — Supplementary Table 1: Characteristics of consecutive patients with INOCA referred to the specialised outpatient clinic [file 12471_2020_1532_MOESM1_ESM.docx]

**Supplementary material**

*Supplementary Table 1*

|  | **All patients**  ***n* = 206** |
| --- | --- |
| Mean age at intake (in years), mean ± SD | 56 ± 9 |
| Female, n (%) | 197 (96%) |
|  |  |
| **Traditional CV risk factors** |  |
| Hypertension | 99 (48%) |
| Hypercholesterolemia | 75 (36%) |
| Smoking |  |
| Current smoker | 15 (7%) |
| Former smoker | 90 (44%) |
| Family history of premature CVD | 131 (64%) |
| Diabetes | 32 (16%) |
| History of obstructive CAD | 46 (22%) |
| History of myocardial infarction | 30 (15%) |
|  |  |
| **Non-traditional CV risk variables, n(%)** |  |
| (Premenopausal) migraine | 81 (39%) |
| Rheumatoid diseases | 45 (22%) |
| Thyroid dysfunction | 36 (18%) |
|  |  |
| **Female-specific CV risk factors, n(%)** |  |
| Hypertensive pregnancy disorders, n=181 | 65 (36%) |
| Multiple spontaneous abortions, n=177 | 41 (23%) |
|  |  |
| **Angina characteristics** |  |
| Symptom duration (in months), mean ± SD | 45 ± 55 |
| Chest pain | 202 (98%) |
| Dyspnoea | 152 (74%) |
| Palpitations | 101 (49%) |
| Radiation of symptoms | 175 (85%) |
|  |  |
| **Diagnostic tests before referral** |  |
| Anatomical: ≥ 2 CAGs or CCTAs | 93 (45%) |
| Functional: ≥ 2 non-invasive ischaemia detection tests | 65 (32%) |
